# Genetic deficiency in neuronal peroxisomal fatty acid β-oxidation causes the interruption of dauer development in *Caenorhabditis elegans*

**DOI:** 10.1038/s41598-017-10020-x

**Published:** 2017-08-24

**Authors:** Saeram Park, Young-Ki Paik

**Affiliations:** 1 Department of Integrated OMICS for Biomedical Science, Yonsei University, 50 Yonsei-ro, Seodaemun-gu, Seoul, 03722 Republic of Korea; 2 Department of Biochemistry, College of Life Science, Yonsei University, 50 Yonsei-ro, Seodaemun-gu, Seoul, 03722 Republic of Korea; 3 Yonsei Proteome Research Center, Yonsei University, 50 Yonsei-ro, Seodaemun-gu, Seoul, 03722 Republic of Korea

## Abstract

Although peroxisomal fatty acid (FA) β-oxidation is known to be critical for animal development, the cellular mechanisms that control the manner in which its neuronal deficiency causes developmental defects remain unclear. To elucidate the potential cellular consequences of neuronal FA metabolic disorder for dauer development, an alternative developmental process in *Caenorhabditis elegans* that occurs during stress, we investigated the sequential effects of its corresponding genetic deficiency. Here, we show that the *daf-*22 gene in peroxisomal FA β-oxidation plays a distinct role in ASK neurons, and its deficiency interrupts dauer development even in the presence of the exogenous ascaroside pheromones that induce such development. Un-metabolized FAs accumulated in ASK neurons of *daf*-*22* mutants stimulate the endoplasmic reticulum (ER) stress response, which may enhance the XBP-1 activity that promotes the transcription of neuronal insulin-like peptides. These sequential cell-autonomous reactions in ASK neurons then activate insulin/IGF-1 signaling, which culminates in the suppression of DAF-16/FOXO activity. This suppression results in the interruption of dauer development, independently of pheromone presence. These findings suggest that neuronal peroxisomal FA β-oxidation is indispensable for animal development by regulating the ER stress response and neuroendocrine signaling.

## Introduction

In animals, fat metabolism influences not only the production of energy and metabolic components but also survival and development. One of the fat metabolic processes, peroxisomal fatty acid (FA) β-oxidation, specifically degrades FAs with very long carbon chains (very long chain FAs; VLCFAs) into short chain FAs (SCFAs). In mammals, this process is especially critical for survival because peroxisome malfunction results in neurodevelopmental disorders that lead to infant mortality (e.g. Zellweger syndrome, X-linked adrenoleukodystrophy)^1^. The elevated accumulation of VLCFAs has been suggested as a cause of these peroxisomal disorders. Peroxisomal FA β-oxidation was recently found to occur in neurons^2, 3^. However, its exact function in neuronal cells is unknown. Moreover, the molecular mechanism by which a deficiency in peroxisomal FA β-oxidation within neurons systemically disrupts development remains to be understood.

*Caenorhabditis elegans* is a free-living nematode that possesses an FA metabolism similar to that of mammals^4^. In particular, peroxisomal FA β-oxidation is required for the biosynthesis of its ascaroside pheromones^5–8^, which induce dauer development. In response to environmental stressors such as high temperature or high population density, the worms excrete increased ascaroside pheromones to alert other larvae to develop into dauer diapause, which is critical for survival in unfavorable conditions^9, 10^. Genetic disruption in peroxisomal FA β-oxidation causes deficiencies in ascaroside biosynthesis and dauer development that are accompanied by developmental abnormalities^7, 11, 12^. However, the exact mechanism by which such developmental defects are caused by a deficiency in peroxisomal FA β-oxidation is not well characterized.

The dauer development of *C*. *elegans* is regulated by neuroendocrine signaling pathways including insulin/insulin-like growth factor-1 (IGF-1) signaling (IIS) and transforming growth factor-β (TGF-β) signaling^13, 14^. Especially in the case of IIS, sensory neurons secrete neuroendocrine signals such as insulin-like peptides (ILPs), the ligands of insulin receptor DAF-2^15^. Then, through the phosphorylation cascade of IIS, activated DAF-2 suppresses the transcription factor DAF-16/FOXO, which is required for dauer development and longevity. To date, the link between FA metabolic disorders (e.g. the accumulation of VLCFAs as a result of defective peroxisomal FA β-oxidation) in neurons and alterations in neuroendocrine signaling (e.g. IIS) that lead to an interruption in dauer development in *C. elegans* also remains unclear.

It was recently revealed that the disturbance of lipid homeostasis can induce the endoplasmic reticulum (ER) stress response^16, 17^. The ER stress response is mediated by three branches: protein kinase RNA-like ER kinase (PEK-1), activating transcription factor 6 (ATF-6), and inositol-requiring-enzyme 1/X-box binding protein-1 (IRE-1/XBP-1)^18^. In the IRE-1/XBP-1 branch, the activation of IRE-1 induces splicing of *xbp-1* mRNA, generating active transcription factor XBP-1, which transcribes several genes including chaperones^19^. The ER stress response, together with metabolic changes, can also be induced by peroxisomal defects, possibly through an unbalanced lipid metabolism^20, 21^. However, in *C. elegans*, it is not well understood whether a deficiency in peroxisomal FA metabolism is linked to ER stress, thereby disrupting developmental plasticity in adverse conditions.

To answer these longstanding questions of how neuronal deficiency in FA β-oxidation causes developmental defects, we investigated the cellular mechanisms that cause the interruption of an alternative developmental process. Here, we show that a deficiency in peroxisomal FA β-oxidation in sensory neurons (i.e. ASK) stimulates the ER stress response, which consequently activates ILP expression and IIS, leading to the prevention of pheromone-induced dauer development.

## Results and Discussion

### Peroxisomal FA β-oxidation occurs in chemosensory neurons, and its deficiency interrupts dauer development in *C. elegans*

**Figure 1:**
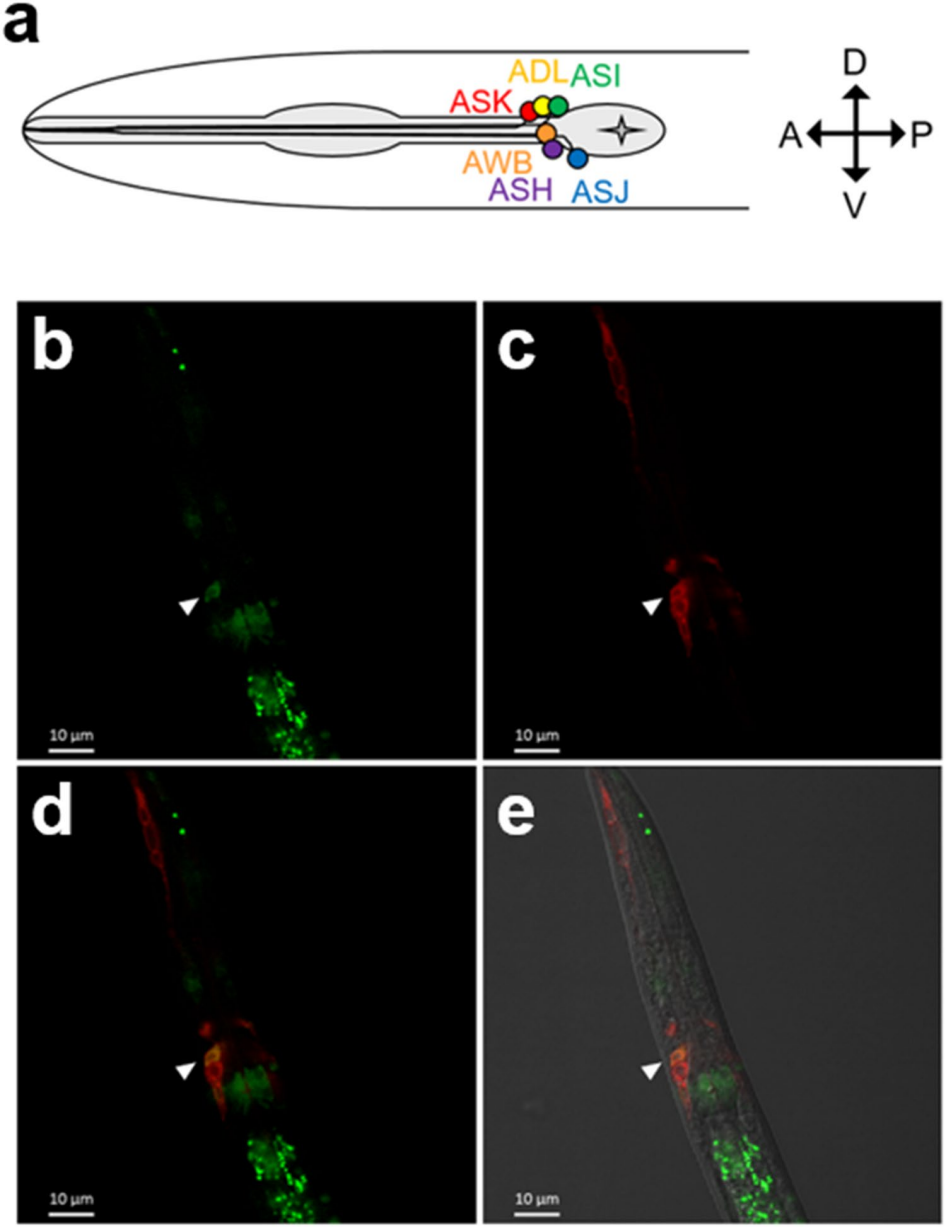
Peroxisomal FA β-oxidation gene *daf-22* expresses in ASK chemosensory neurons. (**a**) A schematic illustration of representative amphid chemosensory neurons (ASK, ADL, ASI, AWB, ASH, and ASJ). Each neuron is annotated. A, anterior; P, posterior; D, dorsal; and V, ventral. (**b–e**) *daf-22p*::GFP::*daf-22* expression in a representative ASK neuron of an L2-stage worm. The ASK neuron is indicated by a solid arrowhead. Images are lateral views of the head region. Scale bar, 10 μm. (**b**) The expression loci of *daf-22p*::GFP::*daf-22* in intestine and neurons. (**c**) DiI staining identifies amphid chemosensory neurons where ASK, ADL and ASI neurons (in order from anterior to posterior) are shown. (**d**) Merged image of *daf-22p*::GFP::*daf-22* expression and DiI-stained neurons. (**e**) Merged image of *daf-22p*::GFP::*daf-22* expression, DiI-stained neurons and differential interference contrast (DIC) is shown for orientation.

Previously, *C. elegans* peroxisomal FA β-oxidation genes (e.g. *acox-1*, *maoc-1*, *dhs-28*, and *daf-22*) were only reported to function in the intestine and hypodermis, where they contribute to ascaroside pheromone biosynthesis^7, 8, 22^. However, our preliminary observations and recent publications by others^2, 3, 7^ led us to suspect that peroxisomal FA β-oxidation may have an as yet unexplored function in neurons. We therefore initially investigated whether genes involved in peroxisomal FA β-oxidation could express in the neurons of *C. elegans*, as reported in mammals. Our examination revealed that *daf-22p*::GFP::*daf-22*, the reporter of the peroxisomal FA β-oxidation gene *daf-22*, was expressed in the neurons of *C. elegans* as well as in the intestine and hypodermis, and its neuronal expression loci were co-localized with ASK neurons (Fig. 1). This neuronal expression was not stage-specific, as *daf-22* was expressed in ASK neurons throughout all developmental stages (Supplementary Fig. S1). Because the ASK neuron is a type of ciliated chemosensory neurons involved in pheromone-sensing and dauer development^23^, this result suggests that peroxisomal FA β-oxidation may have a role in sensory neurons and could contribute to the responses to pheromones in *C. elegans*.

Because *daf-22* was shown to be expressed in chemosensory neurons, we were keen to examine whether its neuronal function systemically influences animal development. As noted above, *C. elegans* larvae can switch their developmental process in response to environmental stressors, and this developmental plasticity can be induced by stress signals such as pheromones. We assessed whether mutant worms deficient in peroxisomal FA β-oxidation, which are defective in ascaroside biosynthesis, would respond to exogenous pheromones and alter their developmental processes to initiate dauer development. Interestingly, when pheromone-induced dauer formation was examined using synthetic ascarosides (Ascr #1, Ascr #2, and Ascr #3) (Supplementary Fig. S2a), fewer than 10% of the peroxisomal FA β-oxidation-deficient mutants (*dhs-28(tm2581)* and *daf-22(ok693)*) entered dauer development in response to exogenous pheromones, whereas 40% to 60% of the wild-type (WT) N2 and mitochondrial FA β-oxidation-deficient mutant *kat-1(tm1037*) worms developed into dauers (Supplementary Fig. S2b). This result indicates that a deficiency in peroxisomal FA β-oxidation specifically interrupts dauer development even in the presence of exogenous ascaroside pheromones. Among those mutants, *daf-22(ok693)* showed the lowest dauer development in the presence of pheromones, so this strain was used as a representative peroxisomal FA β-oxidation–deficient mutant to investigate the manner in which deficiencies in peroxisomal FA β-oxidation interfere with developmental responses to exogenous pheromones.

To determine the manner in which pheromone responsiveness was attenuated, we examined whether either the ciliated chemosensory neuronal structure or pheromone-sensing ability was disrupted by defects in peroxisomal FA β-oxidation. We found that *daf-22(ok693)* worms had normally structured amphid neuronal cells. The amphid neurons were well-stained by DiI dye, indicating that there were no ciliary defects (Supplementary Fig. S2c). Furthermore, *daf-22(ok693)* and WT worms showed identical avoidance in response to exogenous pheromones (Supplementary Fig. S2d), suggesting that pheromone-sensing is normal in this mutant and that defects downstream of pheromone-sensing may interfere with the dauer development. Taken together, these findings confirm that peroxisomal FA β-oxidation is not only involved in ascaroside pheromone biosynthesis^7, 8^, but also specifically required for pheromone responsiveness and developmental decisions in chemosensory neurons (e.g. ASK, ASI, and ASJ)^23, 24^.

### Peroxisomal FA β-oxidation in ASK neurons is essential for pheromone-induced dauer development, but independent of ascaroside pheromone biosynthesis

**Figure 2:**
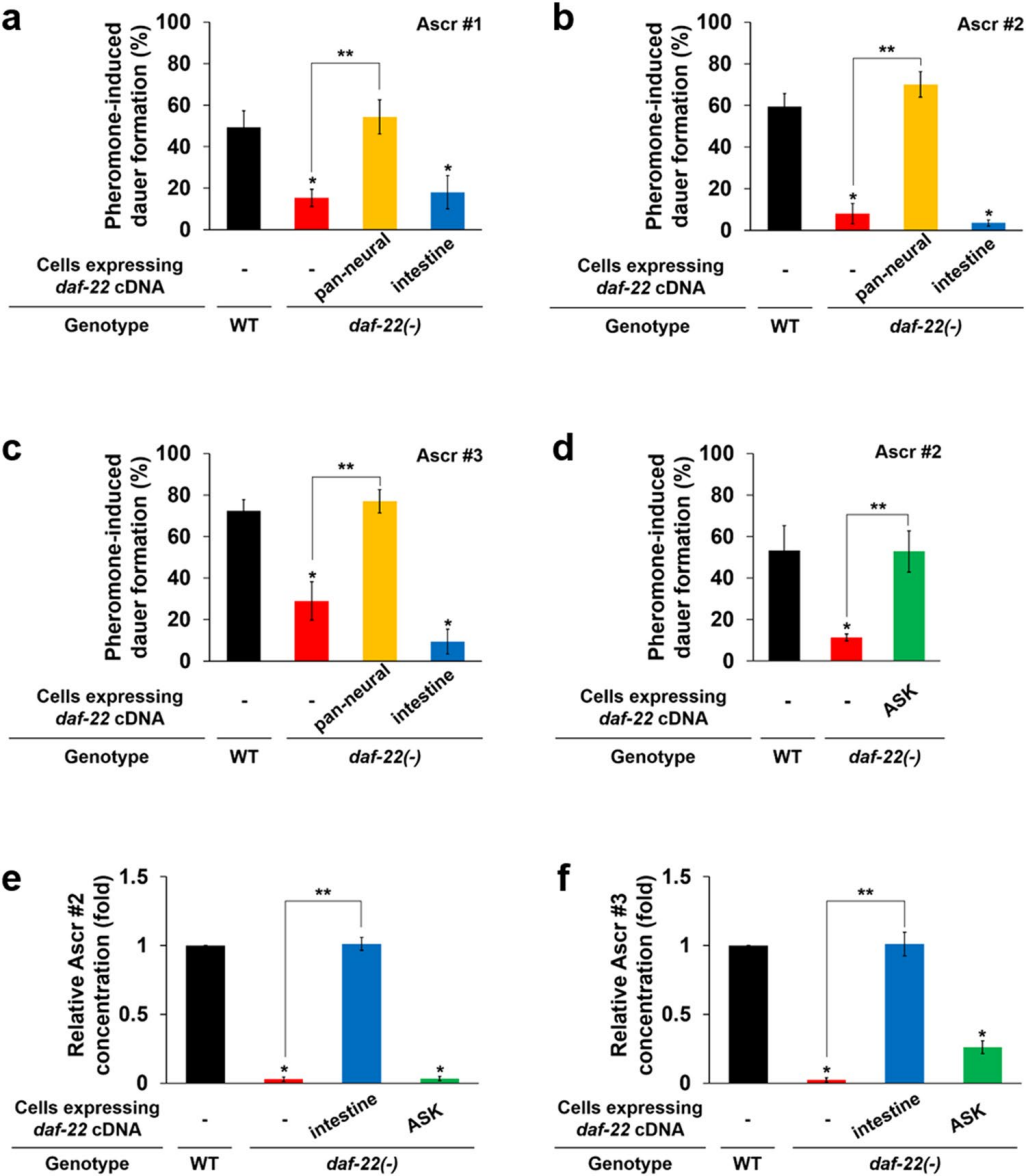
Function of peroxisomal FA β-oxidation gene *daf-22* in ASK neurons is required for pheromone-induced dauer development. (**a**–**c**) Percentage of pheromone-induced dauer formation of tissue-specific (pan-neural- and intestine-specific) *daf-22* rescue worms (Ascr #1 (**a**), Ascr #2 (**b**), and Ascr #3 (**c**)). Data are shown as mean ± SEM of five independent experiments. (**d**) Percentage of pheromone (Ascr #2)-induced dauer formation of ASK neuron-specific *daf-22* rescue worms. Data are shown as mean ± SEM of three independent experiments. (**a–d**) *P < 0.05 compared with WT, **P < 0.05 compared with *daf-22(ok693)* worms, Student’s *t*-test. (**e**,**f**) Relative concentration of worm-body ascaroside pheromones Ascr #2 (**e**) and Ascr #3 (**f**) in intestine- and ASK neuron-specific *daf-22* rescue worms. Data are shown as mean ± SEM of four independent experiments with three technical repeats. *P < 0.001 compared with WT, **P < 0.001 compared with *daf-22(ok693)* worms, Student’s *t*-test. See also Supplementary Table S1.

To examine whether the neuron-specific function of peroxisomal FA β-oxidation is required for pheromone responsiveness and whether interrupted dauer development results from defective pheromone biosynthesis, we next attempted to genetically restore the function of *daf-22* in a tissue-specific manner to examine the recovery of pheromone-induced dauer development. We found that the interrupted pheromone-induced dauer development of *daf-22(ok693)* worms was fully recovered by pan-neural restoration of *daf-22* function (Fig. 2a,b, and c). Interestingly, even though ascaroside biosynthesis was fully restored^22^, the recovery of dauer development in response to pheromones was not observed with an intestine-specific rescue of *daf-22*, suggesting that these two reactions (pheromone biosynthesis and pheromone-induced dauer development) are independent. In other words, neuronal peroxisomal FA β-oxidation has a distinct role in development (in this case, dauer development) in addition to its role in the intestine. Furthermore, and to our surprise, ASK neuron-specific restoration of *daf-22* function seemed sufficient to fully restore pheromone-induced dauer development in the *daf-22(ok693)* worms (Fig. 2d). These results suggest that *daf-22* function in ASK neurons is specifically required for dauer development in response to exogenous pheromones. To the best of our knowledge, this is the first report of the distinct role of FA metabolism in ASK chemosensory neurons in the developmental decisions of *C. elegans*.

A good correlation has been reported between the rate of dauer development and the increased level of ascaroside biosynthesis by peroxisomal FA β-oxidation in *C. elegans* ^10, 12^. We were interested in investigating whether neuronal peroxisomal FA β-oxidation induces dauer development by enhancing pheromone biosynthesis, so we measured the relative quantities of ascarosides in *daf-22* rescue worms (Supplementary Table S1). Notably, we found that intestinally expressed *daf-22* fully restored the biosynthesis of Ascr #2 and Ascr #3 as previously reported^22^, whereas *daf-22* in ASK neurons did not (Fig. 2e,f). This indicates that the synthesis of a quantity of pheromones by peroxisomal FA β-oxidation in ASK neurons is not sufficient to induce dauer development. Rather, the sensory neuron-specific function of peroxisomal FA β-oxidation is essential for pheromone-induced developmental decisions, distinct from its known roles in ascaroside pheromone biosynthesis in the intestine.

### Cellular basis of activation of IIS by peroxisomal FA β-oxidation deficiency, leading to an interruption of dauer development

**Figure 3:**
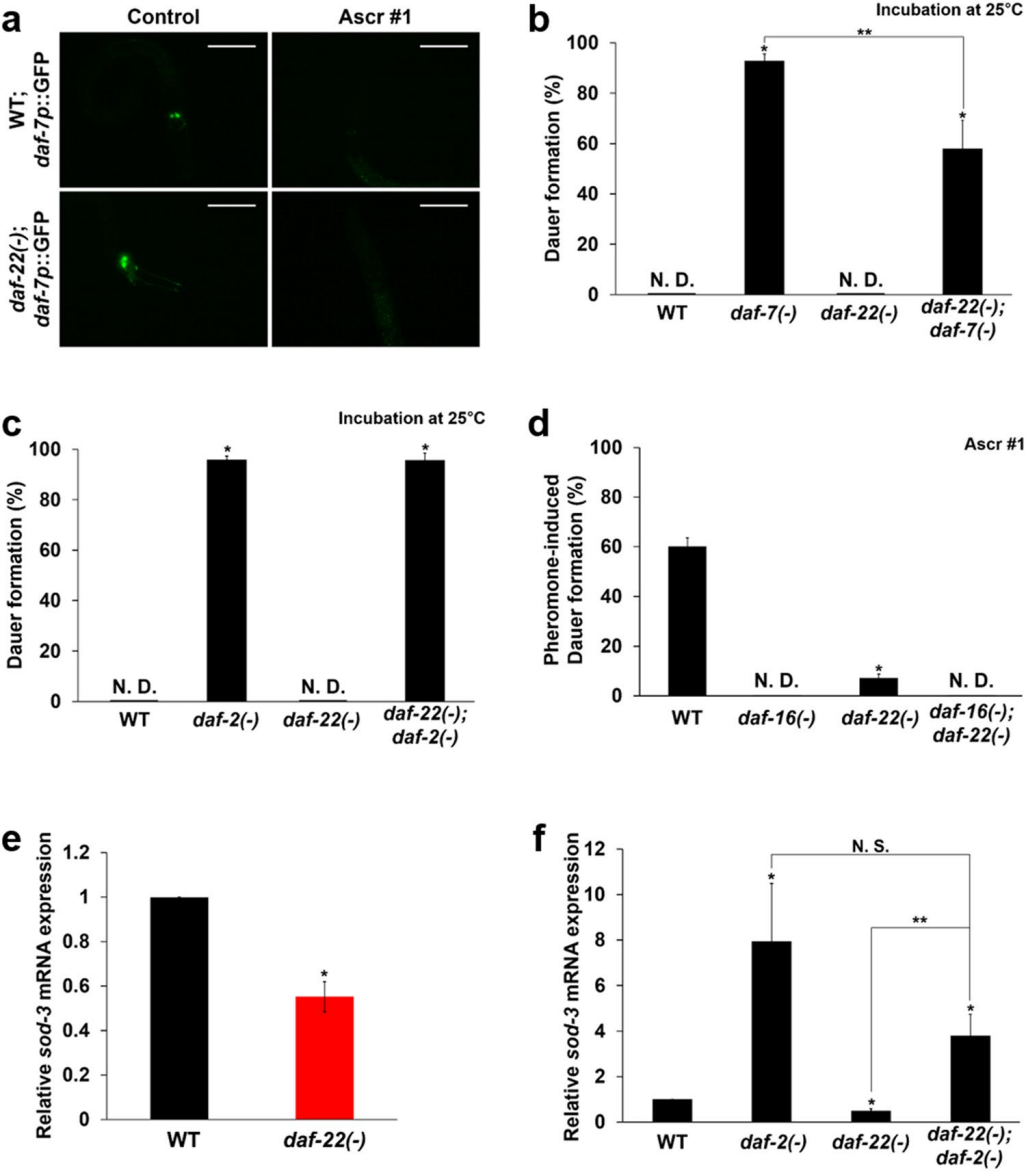
Dauer development in *daf-22(ok693)* worms is dependent on DAF-16 activity. (**a**) Changes of *daf-7p*::GFP expression in response to exogenous pheromone (Ascr #1) in WT and *daf-22(ok693)* worms. Images represent views of the head region of a representative L2-stage worm. Scale bar, 50 μm. See also Supplementary Fig. S3a,b, and Supplementary Table S2. (**b**) Percentage of dauer formation in WT, *daf-7(e1372)*, *daf-22(ok693)*, and *daf-22(ok693)*;*daf-7(e1372)* worms at 25 °C. (**c**) Percentage of dauer formation in WT, *daf-2(e1370)*, *daf-22(ok693)*, and *daf-22(ok693)*;*daf-2(e1370)* worms at 25 °C. (**d**) Percentage of pheromone (Ascr #1)-induced dauer formation in WT, *daf-16(mu86)*, *daf-22(ok693)*, and *daf-16(mu86)*;*daf-22(ok693)* worms. (**b–d**) Data are shown as mean ± SEM of three independent experiments. N.D., not detected, *P < 0.05 compared with WT, **P < 0.05 compared with *daf-7(e1372)* worms, Student’s *t*-test. (**e**) Relative mRNA expression of *sod-3* in *daf-22(ok693)* worms. (**f**) Relative *sod-3* mRNA expression in WT, *daf-2(e1370)*, *daf-22(ok693)* and *daf-22(ok693)*;*daf-2(e1370)* worms. (**e–f**) Data are shown as mean ± SEM of three independent experiments. *P < 0.05 compared with WT, N.S., not significant, and **P < 0.05 compared with *daf-22(ok693)*;*daf-2(e1370)* worms, Student’s *t*-test.

Given that peroxisomal FA β-oxidation in ASK neurons is essential for decisions to enter dauer development, we wished to investigate its effects on the neuroendocrine signaling pathways that regulate dauer development in response to exogenous pheromones. Because it was shown that *daf-22(ok693)* exhibits interrupted dauer development in the presence of pheromones despite its apparently normal neuronal structure and pheromone-sensing ability (see Supplementary Fig. S2), we suspected that there might be a problem with the cellular signaling pathways involved in dauer development (e.g. TGF-β signaling and IIS). To clarify this issue, we first examined TGF-β signaling and found that *daf-7* expression was clearly suppressed in *daf-22(ok693)* mutant worms by exogenous ascarosides and that the expression of *daf-7* is not affected by *daf-22* deficiency (Fig. 3a, Supplementary Fig. S3a,b, and Supplementary Table S2)^25^, confirming that the regulation of TGF-β signaling activity in response to pheromones seemed normal in *daf-22(ok693)*. Moreover, the genetic epistasis between *daf-22* and *daf-7* revealed that dauer development in *daf-22(ok693)* worms was not totally dependent of TGF-β signaling, as the dauer formation of *daf-22(ok693)*;*daf-7(e1372)* was substantially suppressed compared to that of *daf-7(e1372)* (Fig. 3b). Next, we examined whether dauer development in *daf-22(ok693)* worms required DAF-16/FOXO activity in an IIS-dependent manner and found that the defective dauer formation was fully restored by *daf-2* mutation (Fig. 3c), whereas the partial pheromone-induced dauer development of *daf-22(ok693)* worms was completely inhibited by *daf-16* deficiency (Fig. 3d). This result suggests that *daf-22(ok693)* worms’ entry into dauer development is dependent solely on IIS. Because IIS also regulates longevity, we examined whether the lifespan of *daf-22(ok693)* worms depended on IIS. The short lifespan of this mutant was recovered by *daf-2* mutation but decreased by *daf-16* deficiency (Supplementary Fig. S3c,d). Therefore, both the dauer development and lifespan of *daf-22(ok693)* worms appeared likely to be dependent on DAF-16/FOXO activity, confirming the crucial role of IIS-governed DAF-16/FOXO in the animal’s life history. We then assessed change in DAF-16/FOXO activity in *daf-22(ok693)* by measuring the mRNA expression of *sod-3*, a representative target gene of DAF-16/FOXO. We found that the level of DAF-16/FOXO activity was suppressed in *daf-22(ok693)* worms (Fig. 3e). This suppression was substantially restored by *daf-2* genetic deficiency (Fig. 3f), indicating that the attenuation of DAF-16/FOXO activity might be caused by enhanced IIS in *daf-22(ok693)* worms. Together, these results clearly suggest that deficiency in peroxisomal FA β-oxidation leads to abnormally enhanced IIS, resulting in the suppression of DAF-16/FOXO activity.

**Figure 4:**
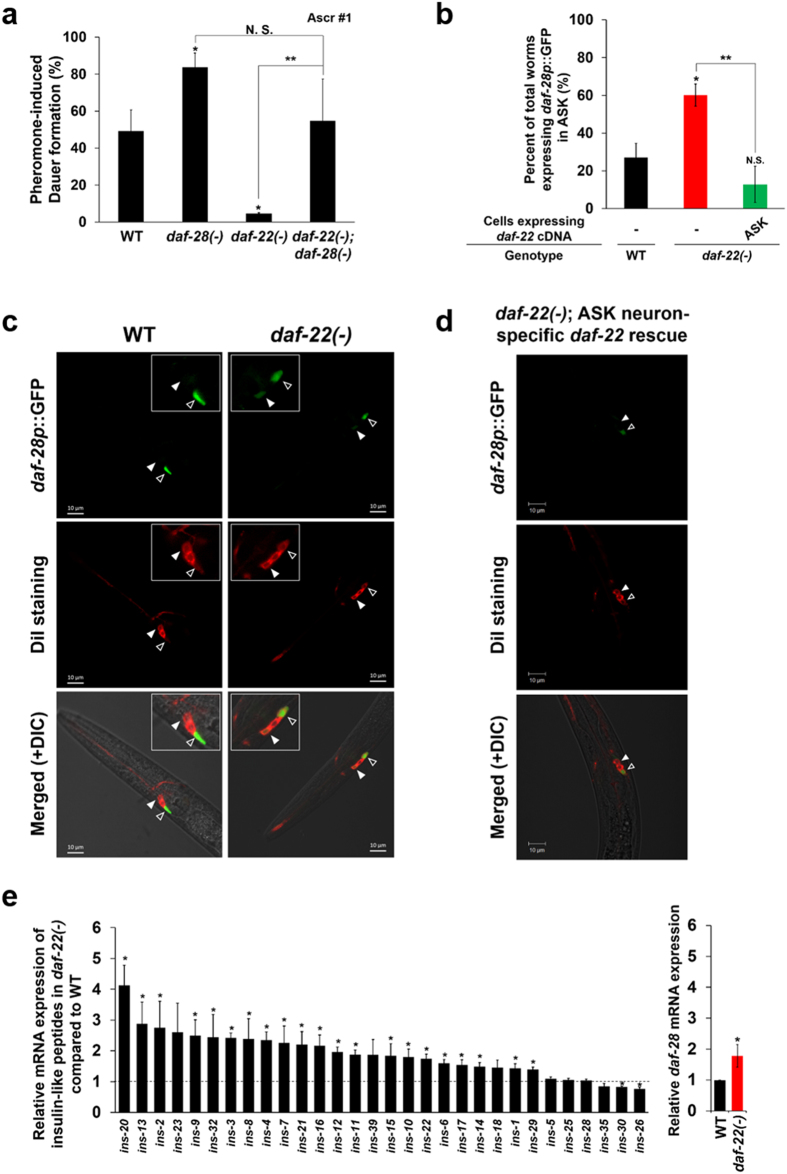
Expression of ILP is increased in *daf-22(ok693)* worms. (**a**) Percentage of pheromone (Ascr #1)-induced dauer formation in WT, *daf-28(tm2308)*, *daf-22(ok693)*, and *daf-22(ok693)*;*daf-28(tm2308)* worms. Data are shown as mean ± SEM of at least two independent experiments. *P < 0.05 compared with WT worms, N.S., not significant, and **P < 0.05 compared with *daf-22(ok693)*;*daf-28(tm2308)* worms, Student’s *t*-test. (**b**) Percentage of total worms expressing *daf-28p*::GFP in ASK neurons in WT, *daf-22(ok693)*, and ASK neuron-specific *daf-22* rescue worms. Data are shown as mean ± SEM of seven independent experiments (WT, n = 59; *daf-22(ok693)*, n = 56; and ASK neuron-specific *daf-22* rescue worms, n = 64). N.S., not significant, *P < 0.005 compared with WT worms, and **P < 0.005 compared with *daf-22(ok693)* worms, Student’s *t*-test. (**c**) *daf-28p*::GFP expression in L2 WT and *daf-22(ok693)* worms. ASK and ASI neurons are indicated by filled and open arrowheads, respectively. Images are lateral views of the head region of a representative L2-stage worm. Inset images are magnified images of ASK, ADL, and ASI neurons (in order from anterior to posterior). Scale bar, 10 μm. (**d**) *daf-28p*::GFP expression in ASK neuron-specific *daf-22* rescue worms. ASK and ASI neurons are indicated by filled and open arrowheads, respectively. Images are lateral views of the head region of a representative worm. Scale bar, 10 μm. (**e**) (*Left*) Changes in relative mRNA expression of ILPs in *daf-22(ok693)* worms compared with WT worms at L2 stage. Dotted line indicates the reference ILP expression in WT worms at L2 stage. Data are shown as mean ± SEM of four independent experiments. (*Right*) Relative *daf-28* mRNA expression in *daf-22(ok693)* compared with WT worms. Data are shown as mean ± SEM of three independent experiments. The figure shown here was drawn based on data presented in Supplementary Fig. S4a for comparison between mRNA levels of ILPs in *daf-22(ok693)*. See also Supplementary Fig. S4a. *P < 0.05 compared with WT worms, Student’s *t*-test.

Several factors are known to enhance insulin signaling, including genetic mutation, ingestion of large amounts of glucose, and fat accumulation. However, the details of the connection between disturbed FA metabolism and the regulation of IIS that influences dauer development are not well known. To assess which factor activates IIS in the peroxisomal FA β-oxidation-deficient mutant *daf-22(ok693)*, we first examined ILPs, the ligands of IIS, in this animal. We found that *daf-28*, one of the dauer formation-related ILPs in *C. elegans* ^26^, might be involved in the prevention of pheromone-induced dauer development in *daf-22(ok693)* worms. The suppressed dauer development of *daf-22(ok693)* was recovered by genetic deficiency of *daf-28* (Fig. 4a). This result implies that *daf-28* is genetically epistatic to *daf-22* for dauer formation (i.e. *daf-28* is downstream of *daf-22*) and that the activation of IIS in *daf-22(ok693)* worms is caused by the enhanced expression of *daf-28*, which is also supported by the increased level of *daf-28* mRNA expression in *daf-22(ok693)* (Supplementary Fig. S4a). Interestingly, increased expression of *daf-28p*::GFP was observed in ASK neurons of *daf-22(ok693)* worms, in addition to the known expression loci ASI and ASJ neurons (Fig. 4b,c). The ratio of *daf-22(ok693)* worms that expressed *daf-28p*::GFP in ASK neurons was more than twice that of WT worms (Fig. 4b). This result is consistent with the recent report of an ectopic expression pattern of *daf-28p*::GFP in mutant worms^27^. Notably, the increased *daf-28p*::GFP expression in ASK neurons of *daf-22(ok693)* worms was suppressed by ASK neuron-specific *daf-22* rescue (Fig. 4b,d), indicating the cell-autonomous regulation of *daf-28* expression by peroxisomal FA β-oxidation. As anticipated, *daf-22p*::GFP and *daf-28p*::DsRed were also co-expressed in head neurons (Supplementary Fig. S4b–e), which also implies their close cooperation in developmental decisions in this nematode. Together, these results suggest that neuronal peroxisomal FA β-oxidation is required for the cell-autonomous regulation of neuronal ILP *daf-28* expression, which may enhance IIS and interrupt dauer development.

Although *daf-28* is one of the major ILPs that regulate dauer development^26^, we cannot exclude the possibility that other ILPs capable of activating IIS are also enhanced in neurons by the deficiency in peroxisomal FA β-oxidation. Therefore, to examine whether the expression of other ILPs can also be altered in *daf-22(ok693)*, we screened the relative mRNA expression of 30 ILPs that express in head neurons^15, 28^ at L2, which is the developmental stage of dauer entry. We found that the transcription of 21 of the 30 ILPs examined was significantly increased in *daf-22(ok693)* compared with WT worms (Fig. 4e), suggesting that these ILPs may act in concert with *daf-28* to activate IIS in *daf-22(ok693)* worms^28^. Therefore, it is plausible to propose a composite effect by which most of these increased ILPs may enhance IIS activity, which in turn prevents pheromone-induced dauer development.

### Deficiency in peroxisomal FA β-oxidation activates the ER stress response

**Figure 5:**
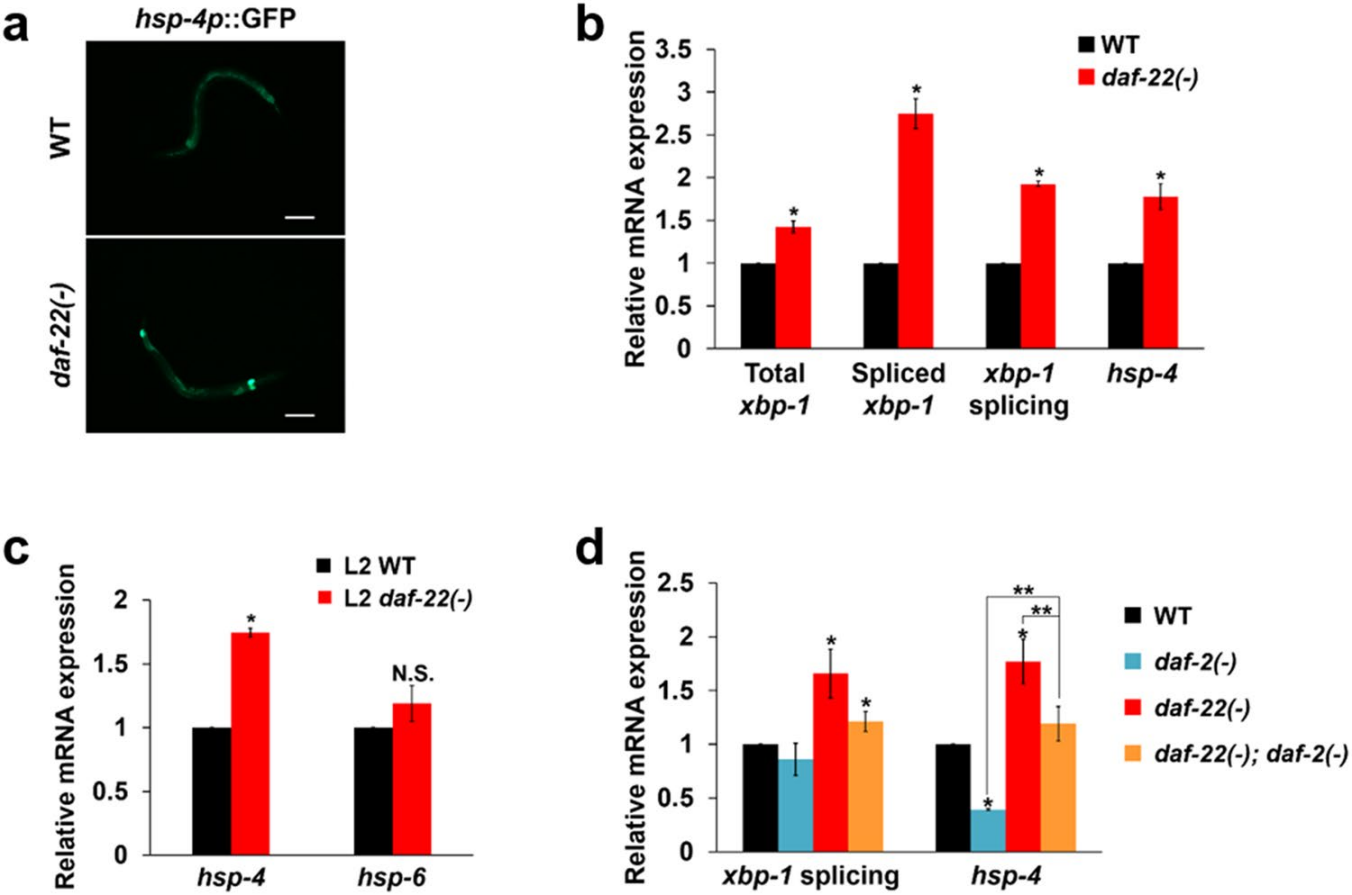
ER stress response is increased in *daf-22(ok693)* worms. (**a**) *hsp-4p*::GFP expression in WT and *daf-22(ok693)* worms. Scale bar, 100 μm. (**b**) Relative abundance of total and spliced *xbp-1* transcript, *xbp-1* splicing (spliced *xbp-1*/total *xbp-1* transcript), and *hsp-4* mRNA expression in *daf-22(ok693)* worms compared with WT worms. (**c**) Relative mRNA expression of *hsp-4* and *hsp-6* in L2 *daf-22(ok693)* worms compared with WT worms. (**d**) Relative abundance of *xbp-1* splicing (spliced *xbp-1*/total *xbp-1* transcript) and *hsp-4* mRNA expression in WT, *daf-2(e1370)*, *daf-22(ok693)*, and *daf-22(ok693)*; *daf-2(e1370)* worms. (**b–d**) Data are shown as mean ± SEM of three independent experiments. N.S., not significant, *P < 0.05 compared with WT, **P < 0.05 compared with *daf-22(ok693)*;*daf-2(e1370)* worms, Student’s *t*-test.

Next, we were keen to elucidate the potential molecular mechanism that underlies the activation of neuronal ILPs associated with a deficiency in peroxisomal FA β-oxidation. Given that ER stress is closely correlated with peroxisomal defects^20, 21^, we first examined whether the expression of ER stress response-related genes can be altered by deficiency in peroxisomal FA β-oxidation. To our surprise, *hsp-4* expression and *xbp-1* mRNA splicing, both indicators of the ER stress response, were substantially elevated in *daf-22(ok693)* worms (Fig. 5a,b). In contrast, the expression of genes representing mitochondrial stress (*hsp-6*) and cytosolic stress (*hsp-16.2*) remained unchanged in *daf-22(ok693)* at the L2 stage of dauer entry (Fig. 5c and Supplementary Fig. S5a). These results suggest that deficiency of peroxisomal FA β-oxidation specifically activates the ER stress response, which may then interrupt entry into the dauer stage at L2. Despite the activation of the ER stress response, *daf-22(ok693)* worms displayed a normal unfolded protein response (UPR) to proteotoxic stress induced by tunicamycin (Supplementary Fig. S5b). Considering the similar phenomena seen in other peroxisomal FA β-oxidation-deficient mutants such as *maoc-1(hj13)* and *dhs-28(tm2581)* (Supplementary Fig. S5c), it can be inferred that the ER stress response is caused more specifically by an increased level of VLCFAs and subsequent imbalance of lipid homeostasis as previously reported^7^. Because it was also reported that active DAF-16/FOXO reprograms the ER stress response regulators and lowers *hsp-4* expression^29^, the ER stress response might be enhanced due to the suppressed DAF-16/FOXO activity in *daf-22(ok693)* worms. However, the increased *hsp-4* mRNA expression in *daf-22(ok693)* worms appeared to be independent of IIS (Fig. 5d), raising the possibility that a deficiency in peroxisomal FA β-oxidation itself could directly induce the ER stress response and the subsequent activation of XBP-1, which might also be a main cause of enhanced neuronal ILP expression and interrupted dauer development in the presence of exogenous pheromones.

### XBP-1 activity, induced by the ER stress response, is involved in cell-autonomous stimulation of ILP expression by a deficiency in peroxisomal FA β-oxidation

**Figure 6:**
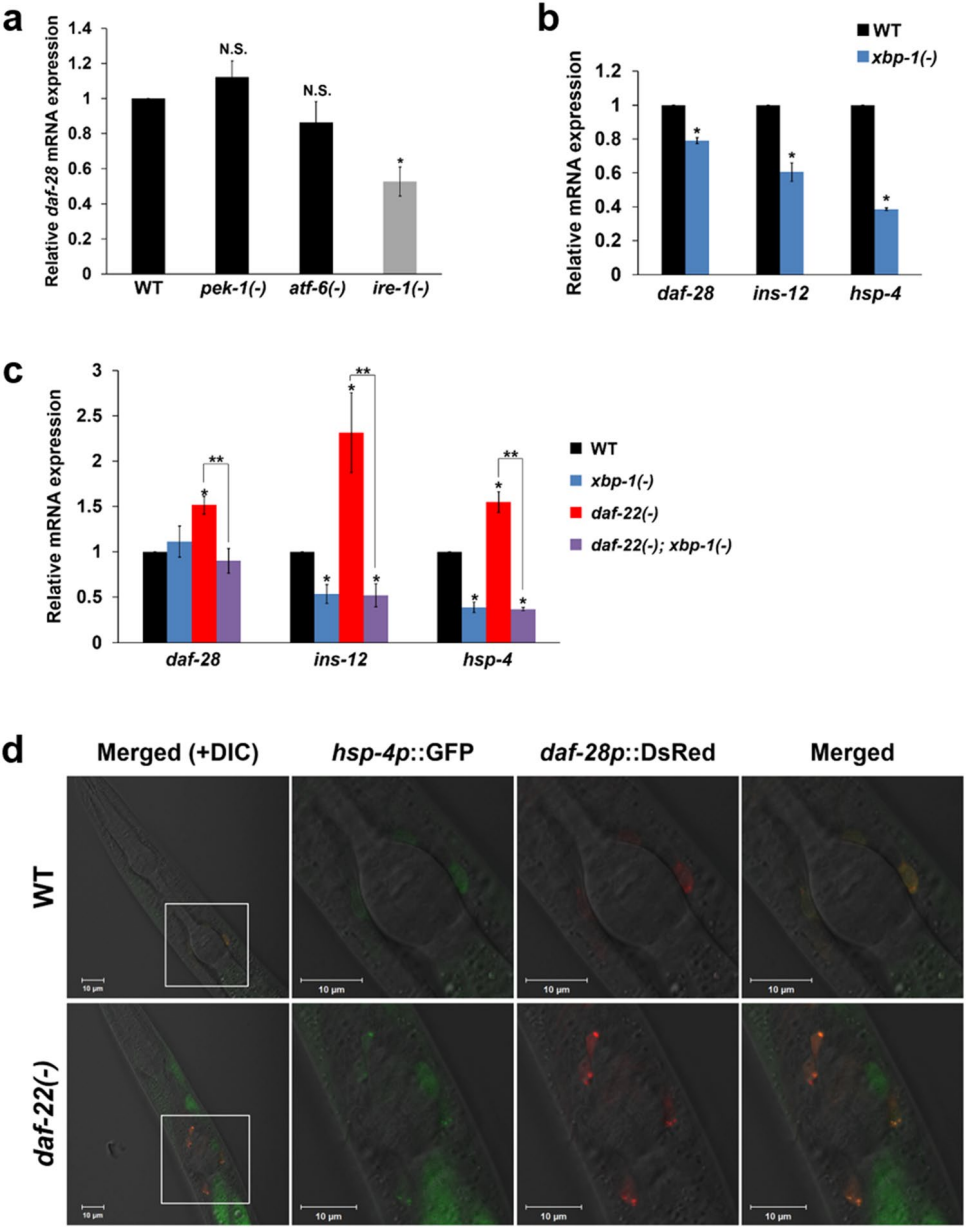
XBP-1 activity stimulated by the increased ER stress response enhances ILP expression in *daf-22(ok693)* worms. (**a**) Relative *daf-28* mRNA expression in the ER stress response mutants, *pek-1(ok275)*, *atf-6(ok551)*, and *ire-1(ok799)* worms at L2 stage. (**b**) Relative mRNA expression of *daf-28*, *ins-12*, and *hsp-4* in L2 *xbp-1(zc12)* mutants compared with WT worms. See also Supplementary Fig. S6b. (**a,b**) Data are shown as mean ± SEM of three independent experiments. N.S., not significant, *P < 0.05 compared with WT worms, Student’s *t*-test. (**c**) Relative mRNA expression of *daf-28*, *ins-12*, and *hsp-4* in L2 WT, *xbp-1(zc12)*, *daf-22(ok693)*, and *daf-22(ok693)*; *xbp-1(zc12)* worms. Data are shown as mean ± SEM of two independent experiments. *P < 0.05 compared with WT, **P < 0.05 compared with *daf-22(ok693)* worms, Student’s *t*-test. (**d**) Expression of *hsp-4p*::GFP and *daf-28p*::DsRed in head neurons of WT and *daf-22(ok693)* worms. Images are lateral views of the head region of a representative worm. The three images on the right are magnified images of the boxed areas in the left-most images of WT and *daf-22(ok693)* worms, respectively. Scale bar, 10 μm.

Next, we investigated whether each branch of ER stress response is involved in stimulating the expression of ILP genes by determining changes in *daf-28* mRNA expression in mutant worms deficient in the ER stress response, *pek-1(ok275)*, *atf-6(ok551)*, and *ire-1(ok799)* worms at L2 stage. Our data show that *ire-1* branch appears to be involved in stimulation of ILP expression because *daf-28* expression was specifically dependent on this branch (Fig. 6a). Because IRE-1 activates transcription factor XBP-1, which is also involved in transcription of IGF1 in zebrafish^30^, we tested whether active XBP-1 can also directly promote the expression of ILP genes in *C. elegans*. We hypothesized that stimulation of ILP gene expression by XBP-1 may be due to its binding to promoter regions of ILPs. Because XBP-1 usually binds to its binding motifs in the promoters of target genes^31^, we closely investigated the upstream sequences of those ILP genes that were significantly increased in *daf-22(ok693)* worms. Notably, among the 15 ILPs that contain several XBP-1 binding motifs in their promoter regions (Supplementary Fig. S6a), the mRNA expression of *daf-28* and *ins-12* seemed more dependent on XBP-1 activity (Fig. 6b and Supplementary Fig. S6b). Moreover, the induction of *daf-28* and *ins-12* expression in *daf-22(ok693)* worms was significantly suppressed by *xbp-1* deficiency (Fig. 6c), indicating that active XBP-1 is required for increased expression of ILPs (e.g. *daf-28* and *ins-12*) in *daf-22(ok693)*. To test whether the enhanced ER stress response could increase *daf-28* expression in a cell-autonomous manner in neurons, we examined the expression loci of *hsp-4* and *daf-28*. Our data showed that *hsp-4* and *daf-28* are co-expressed in several neurons (Fig. 6d), indicating that increased ER stress response in *daf-22(ok693)* can enhance the expression of ILPs including *daf-28* in a cell-autonomous manner. Because the *daf-28* expression in ASK sensory neurons increased in *daf-22(ok693)* worms, we propose that the enhanced ER stress response in ASK neurons caused by a deficiency in peroxisomal FA β-oxidation may stimulate the expression of *daf-28* within these cells, leading to an interruption of dauer development in response to exogenous pheromones. This finding is supported by the fact that XBP1 activity is required for proinsulin processing and secretion in pancreatic β-cells in mammals^32^ and that a chronic mild ER stress response is involved in the activation of insulin signaling^33^.

**Figure 7:**
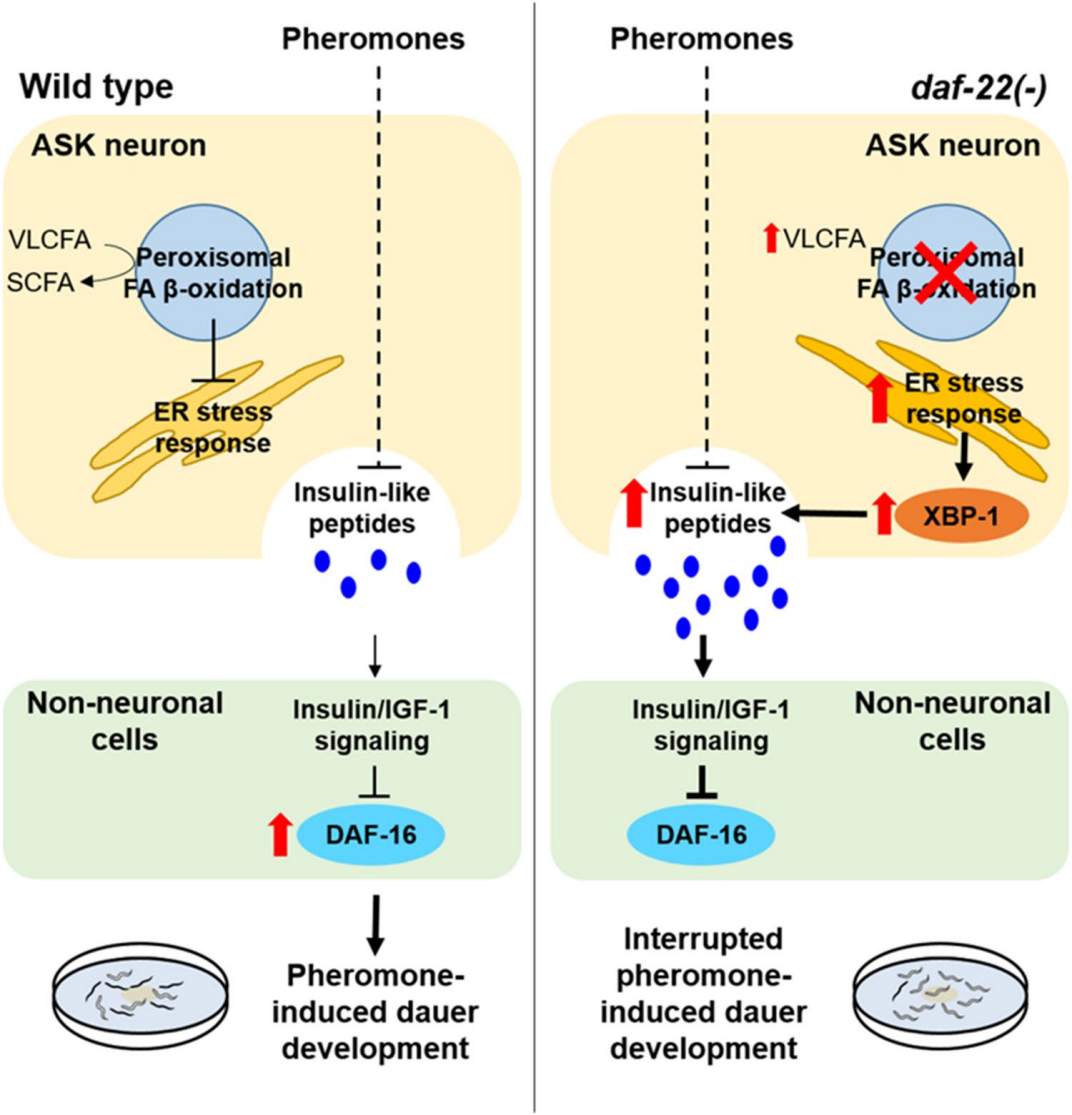
Proposed model for cellular mechanisms causing interruption of alternative developmental process by defective peroxisomal FA β-oxidation in ASK neurons in *C. elegans*. In WT animals, exogenous pheromones representing stressful growth conditions can suppress the expression of neuronal insulin-like peptides (ILPs), diminishing insulin/IGF-1 signaling (IIS) in non-neuronal cells. This causes activation of DAF-16/FOXO, which is required for worms to enter dauer development in response to pheromones. However, in *daf-22(ok693)* mutant worms, deficiency of peroxisomal FA β-oxidation in ASK neurons causes an increase in the levels of VLCFAs and a disturbance in cellular lipid homeostasis, which may subsequently activate the ER stress response and its downstream transcription factor XBP-1. In turn, activated XBP-1 stimulates the transcription of neuronal ILPs, such as *daf-28* and *ins-12*, which would consequently enhance IIS of non-neuronal cells to suppress DAF-16/FOXO. Therefore, dauer development of *daf-22(ok693)* is interrupted even in the presence of exogenous pheromones. Bold arrows and red arrows indicate activation, and the symbol ⊥ indicates inhibition.

In summary, our study reveals cellular mechanisms that cause the interruption of the alternative developmental process in *C. elegans* by a deficiency in peroxisomal FA β-oxidation in sensory neurons, through the sequential actions of multiple molecular components including the ER stress response and neuroendocrine signaling (i.e. IIS) activity (Fig. 7). Based on our results, we propose the following sequence involving the consequence of defective peroxisomal FA β-oxidation in *C. elegans*. Peroxisomal FA β-oxidation plays a distinct role in ASK chemosensory neurons, and this neuronal function is particularly important for dauer development of *C. elegans* in response to exogenous pheromones (Figs 1 and 2). A deficiency in peroxisomal FA β-oxidation (due to genetic mutation of *daf-22*) in ASK neurons may cause the accumulation of excessive un-metabolized VLCFAs^7^, which further activates the ER stress response and subsequent XBP-1 activity (Fig. 5). This enhanced XBP-1 promotes the transcriptional expression of neuronal ILPs (e.g. *daf-28* and *ins-12*) (Fig. 6), and the elevated levels of ILPs (e.g. increased *daf-28* expression in ASK neurons where *daf-22* functions) then stimulate IIS in *daf-22(ok693)* (Fig. 4). Eventually, the enhanced IIS attenuates DAF-16/FOXO activity and interrupts the dauer development of *daf-22(ok693)*, even in the presence of exogenous pheromones (Fig. 3). Although this proposal deserves further validation, our results may contribute to answering the enduring question of how metabolic disorders of FAs in neurons (e.g. accumulation of VLCFAs due to a deficiency in peroxisomal FA β-oxidation) enhance IIS and thereby interrupts DAF-16/FOXO–governed dauer development. Hence, it is worth investigating whether there is a connection among peroxisomal disorders in neurons, alterations in neuroendocrine signaling, and development in mammals, including humans.

## Methods

### *C. elegans* strains and maintenance

All animals were cultured as previously described^34^. *C. elegans* strains were maintained on nematode growth medium (NGM) agar plates, fed with *Escherichia coli* strain OP50 grown in 2xYT, and cultured at 15 °C or 20 °C. The strains used in this study and strain maintenance are described in the Supplementary Methods.

### Cloning and transgenic-line construction

For the experiments involving pan-neural or ASK neuron-specific *daf-22* rescue, transgenes were constructed by polymerase chain reaction (PCR) fusion as previously described^35^. For detailed procedures of transgene construction and transgenic-line construction, see the Supplementary Methods.

### Pheromone-induced dauer formation assay

A pheromone-induced dauer formation assay using ascarosides was performed as previously described^5^ with minor modifications. Synthetic ascarosides (Ascr #1 [daumone 1, ascaroside C7], Ascr #2 [daumone 2, ascaroside C6], and Ascr #3 [daumone 3, ascaroside C9]) were chemically synthesized in our laboratory as previously described^5, 6^ and were dissolved in absolute ethanol. Ascaroside-containing plates were prepared with NGM (without peptone) into which ascaroside solution was mixed at a final concentration of 38 μM. Heat-killed *E. coli* OP50 (160 μg) was added to the center of each plate and dried to provide a limited source of food. Ten to fifteen gravid adult worms were transferred to the ascaroside-containing plates and incubated for 5 hours to lay eggs. After incubation, the adult worms were removed, and the eggs on the plates were incubated at 25 °C for 96 hours. The ratio between dauer and non-dauer (L4 or adult) worms was calculated. When using transgenic worms, only the offspring of transgenic animals with roller phenotypes were scored as dauers or non-dauers. When observing *daf-7p*::GFP expression, a standard concentration (380 μM) of the ascarosides and equivalent amounts of absolute ethanol as the control were used. For dauer formation assays not using ascarosides, NGM plates were used, and the assay was performed at 25 °C.

### Ascaroside pheromone quantification

To analyze the worm-body ascarosides, age-synchronized worms were cultured on NGM plates (without peptone) containing *E*. *coli* strain OP50 (at a concentration of OD_600_ = 2.5) at 20 °C. To analyze the quantity of ascarosides, 40 young adult worms were collected and transferred to 20 µl of distilled water in 1.5-ml conical tubes. The samples were stored at −70 °C before analysis with liquid chromatography–tandem mass spectrometry (LC-MS/MS). The ascarosides in the worm bodies were quantified by the PheroQu (pheromone quantitation) method, as previously described^10, 22^. To identify the ascaroside pheromones, the ascarosides were extracted from the worm samples, separated by ultra-performance liquid chromatography (UPLC) and then monitored with a positive electrospray ionization detector in the multiple-reaction monitoring (MRM) mode using stable deuterated isotope-containing ascarosides as internal standards.

### DiI staining and microscopy

DiI staining was performed to visualize ciliated chemosensory neurons as previously described (www.wormatlas.org/EMmethods/DiIDiO.htm) with minor modifications. Briefly, DiI (1,1′-dilinoleyl-3,3,3′,3′-tetramethylindocarbocyanine perchlorate (FAST DiI™ oil; DiIΔ9 12 -C18(3), CIO4), Molecular Probes) stock solution was prepared at 2 mg/ml concentration in dimethyl formamide and stored at −20 °C. The stock solution was diluted to 1:200 in M9, and 150 μl of solution was put in a glass tube into which L2-synchronized worms were transferred. After staining for 2 hours at 20 °C, the worms were washed with M9 and transferred to a fresh NGM plate to crawl on a bacterial lawn for 1 hour to de-stain. DiI-stained or GFP-expressing worms were anaesthetized by a droplet of 50 mM sodium azide on 3% agar pads on the slide glasses and were visualized with microscopy. Worms were visualized using a confocal microscope LSM 700 (Carl Zeiss) with a Fluorescence Alexa 488 and rhodamine filter. Images were analyzed using ZEN 2.1 software (Carl Zeiss). GFP expression was also observed at low magnification using an Axio Imager A1 microscope (Carl Zeiss). Images were analyzed and relative fluorescence intensity was measure using ImageJ software (NIH).

### Quantitative real-time polymerase chain reaction (qRT-PCR)

Age-synchronized worms were grown on NGM (with *E. coli* OP50 at a concentration of OD_600_ = 2.5 to control the amount of food) at permissive temperature (15 °C or 20 °C) until the L2 (12 hours from L1) or young adult (approximately 60 hours from L1) stage and were then harvested by S-basal. Total RNA was isolated from the worms using TRIzol Reagent (Thermo Fisher Scientific) and purified using the RNeasy kit (QIAGEN). The RNA was reverse-transcribed using a Transcriptor First Strand cDNA Synthesis Kit (Roche) with oligo-dT priming. qRT-PCR was performed using iQ SYBR Green Supermix (Bio-Rad Laboratories) according to the manufacturer’s instructions. The reaction products were analyzed using a CFX Connect™ Real-Time PCR Detection System (Bio-Rad Laboratories). Relative mRNA expression was determined using the ΔΔC_T_ method, and the mRNA expression of *act-1* was used as a reference to normalize the results.

### Pheromone drop assay

The repulsive chemotaxis response to ascaroside pheromones was measured as previously described^36^. Ascr #2 was dissolved in M13 buffer (30-mM Tris-HCl, pH 7.0, 100-mM NaCl, 10-mM KCl) to a final concentration of 10 mM and serially diluted to 100 μM and 10 μM. Forty young adult animals were transferred to unseeded NGM plates and preconditioned for 30 minutes. A droplet of ascaroside solution was applied to a forward-moving worm and the behavioral response of the worm was monitored. Worms showing a long reversal, which is a backward movement equal to or longer than the half-length of the worm, were counted. As a control, the same assay was performed with a drop of M13 buffer. The chemotaxis index (C.I.) was calculated as ([number of animals making long reversal in response to M13 buffer] − [number of animals making long reversal in response to ascaroside solution])/(total number of animals tested).

### Lifespan analysis

Adult lifespans were measured at 20 °C as previously described^37^. 5′-fluorodeoxyuridine (FUDR) (Sigma-Aldrich, 0.1 mg/ml) was used to prevent the growth of progeny.

### Tunicamycin treatment

Tunicamycin (Sigma-Aldrich) stock solution was prepared at a 5 mg/ml concentration in dimethyl sulfoxide (DMSO), stored at −20 °C, and added to NGM at final concentrations of 2 μg/ml and 5 μg/ml. For the control condition (0 μg/ml of tunicamycin), equivalent amounts of DMSO were added to NGM.

### Statistics

The error bars of all results represent the standard error of the mean (SEM), and Student’s *t*-test was used where indicated.

## Electronic supplementary material

Supplementary Information
