# Supplementary material for: Genetic deficiency in neuronal peroxisomal fatty acid β-oxidation causes the interruption of dauer development in Caenorhabditis elegans
Source: Sci Rep. 2017 Aug 24;7:9358. doi: 10.1038/s41598-017-10020-x (PMC5571181; doi:10.1038/s41598-017-10020-x)
Supplement: Supplementary file 1 — Supplementary Information [file 41598_2017_10020_MOESM1_ESM.pdf]

SREP-17-17803A. (Supplementary Information-Clean Version).

Genetic deficiency in neuronal peroxisomal fatty acid β-oxidation causes the interruption of dauer development in *Caenorhabditis elegans*

Saeram Park[1] and Young-Ki Paik[1,2,3,*]

[1]Department of Integrated OMICS for Biomedical Science, Yonsei University, 50 Yonsei-ro, Seodaemun-gu, Seoul, 03722, Republic of Korea

[2]Department of Biochemistry, College of Life Science, Yonsei University, 50 Yonsei-ro, Seodaemun-gu, Seoul, 03722, Republic of Korea

[3]Yonsei Proteome Research Center, Yonsei University, 50 Yonsei-ro, Seodaemun-gu, Seoul, 03722, Republic of Korea

[*]Correspondence and requests for materials should be addressed to Y.-K.P. (email: paikyk@yonsei.ac.kr)

SUPPLEMENTARY FIGURES AND LEGENDS

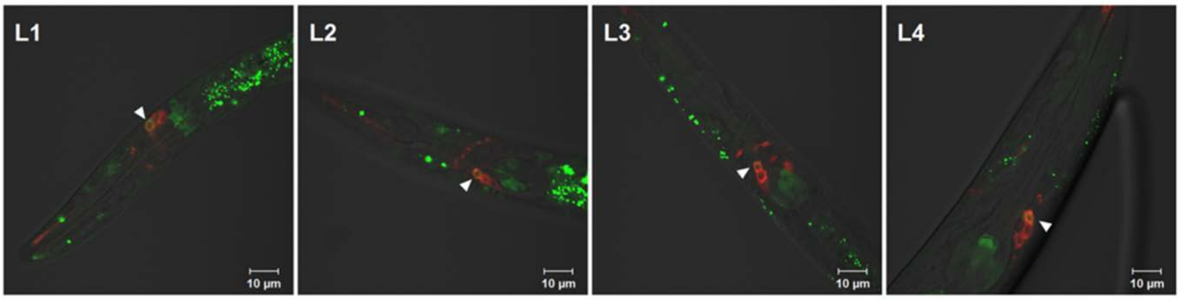

Supplementary Fig. S1. Peroxisomal FA β-oxidation gene *daf-22* expresses in ASK neurons.

daf-22p::GFP::*daf-22* expresses in ASK neurons throughout all the developmental stages. Images are lateral views of the head region of the representative worms in L1, L2, L3, and L4 developmental stages, respectively. Merged images of *daf-22p*::GFP::*daf-22* expression, DiI-stained amphid chemosensory neurons and DIC are shown. The DiI-stained cells are ASK, ADL, and ASI neurons (in order from anterior to posterior). ASK neurons are indicated by solid arrowheads. Scale bar, 10 μm.

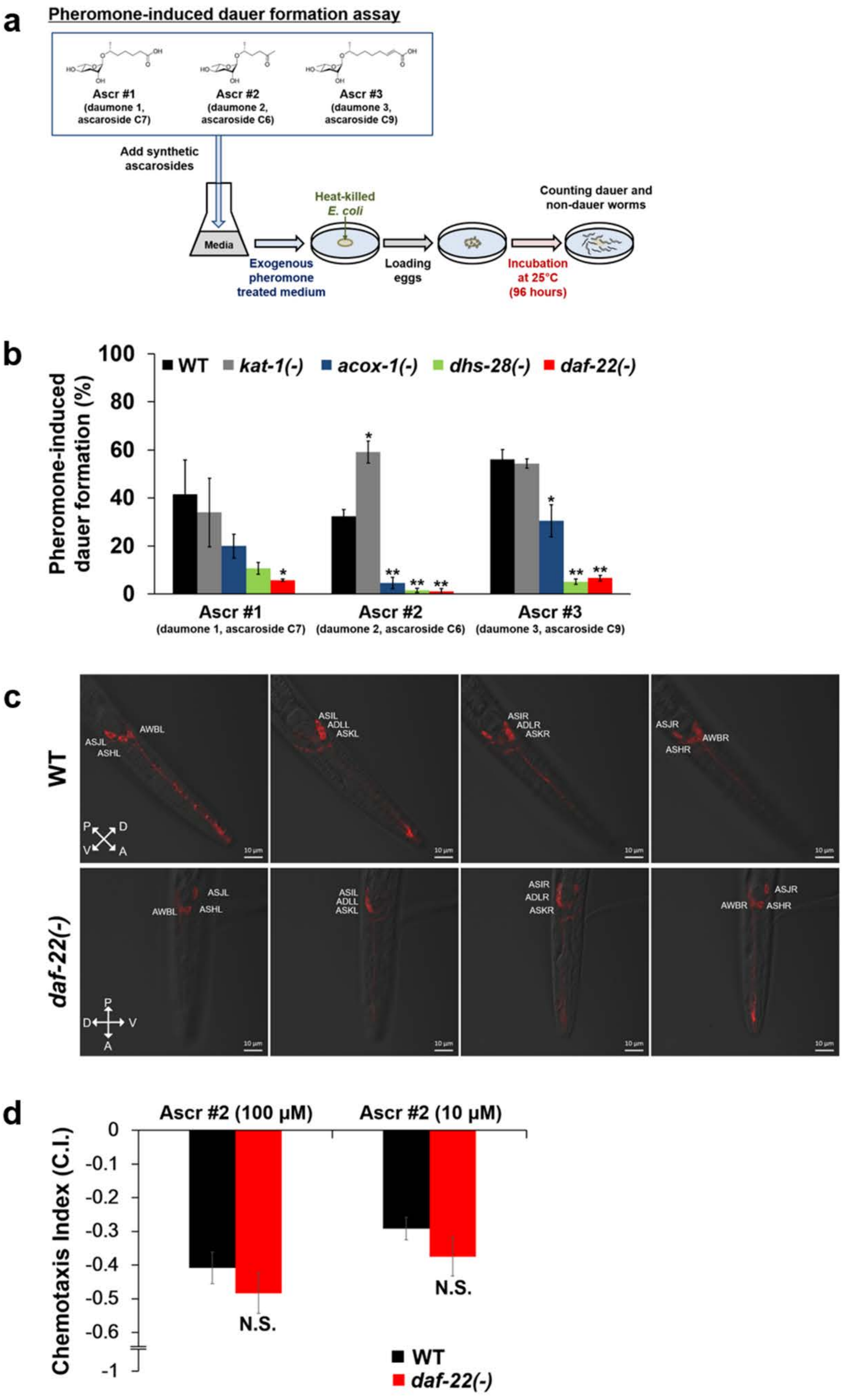
a
Pheromone-induced dauer formation assay
Ascr #1 (daumone 1, ascaroside C7)
Ascr #2 (daumone 2, ascaroside C6)
Ascr #3 (daumone 3, ascaroside C9)
Add synthetic ascarosides
Media
Exogenous pheromone treated medium
Heat-killed E. coli
Loading eggs
Incubation at 25°C (96 hours)
Counting dauer and non-dauer worms
b
WT
kat-1(-)
acox-1(-)
dhs-28(-)
daf-22(-)
Pheromone-induced dauer formation (%)
100
80
60
40
20
0
Ascr #1 (daumone 1, ascaroside C7)
Ascr #2 (daumone 2, ascaroside C6)
Ascr #3 (daumone 3, ascaroside C9)
c
WT
daf-22(-)
AWBL
ASJL
ASHL
ASIL
ADLL
ASKL
ASIR
ADLR
ASKR
ASJR
AWBR
ASHR
P
D
V
A
10 μm
d
Ascr #2 (100 μM)
Ascr #2 (10 μM)
Chemotaxis Index (C.I.)
0
-0.1
-0.2
-0.3
-0.4
-0.5
-0.6
-1
N.S.
N.S.
WT
daf-22(-)

Supplementary Fig. S2. Deficiency in peroxisomal FA β-oxidation interrupts pheromone-induced dauer development without defects in neuronal structure or pheromone-sensing ability.

(**a**) Schematic illustration of pheromone-induced dauer formation assay.

(**b**) Pheromone-induced dauer formation in WT, mitochondrial FA β-oxidation-deficient *kat-1(tm1037)* mutants, peroxisomal FA β-oxidation-deficient mutant worms *acox-1(ok2257), dhs-28(tm2581),* and *daf-22(ok693)* was examined using Ascr #1, Ascr #2, and Ascr #3 (38 μM). Data are shown as mean ± SEM of three independent experiments. * $P < 0.05$ and ** $P < 0.001$ compared with WT worms, Student's *t*-test.

(**c**) DiI staining of amphid chemosensory neurons in WT and *daf-22(ok693)* worms. Images are lateral views of the head region of a representative L2-stage worm. DiI dye-stained amphid neurons (ASK, ADL, ASI, AWB, ASH, and ASJ) are annotated. A, anterior; P, posterior; D, dorsal; and V, ventral. Scale bar, 10 μm.

(**d**) Repulsion responses to ascaroside (Ascr #2, 100 μM and 10 μM) in WT and *daf-22(ok693)* worms were measured using pheromone drop assay and represented as chemotaxis index. Data are shown as mean ± SEM of three independent experiments of 40 animals for each assay. N.S., not significant compared with WT worms.

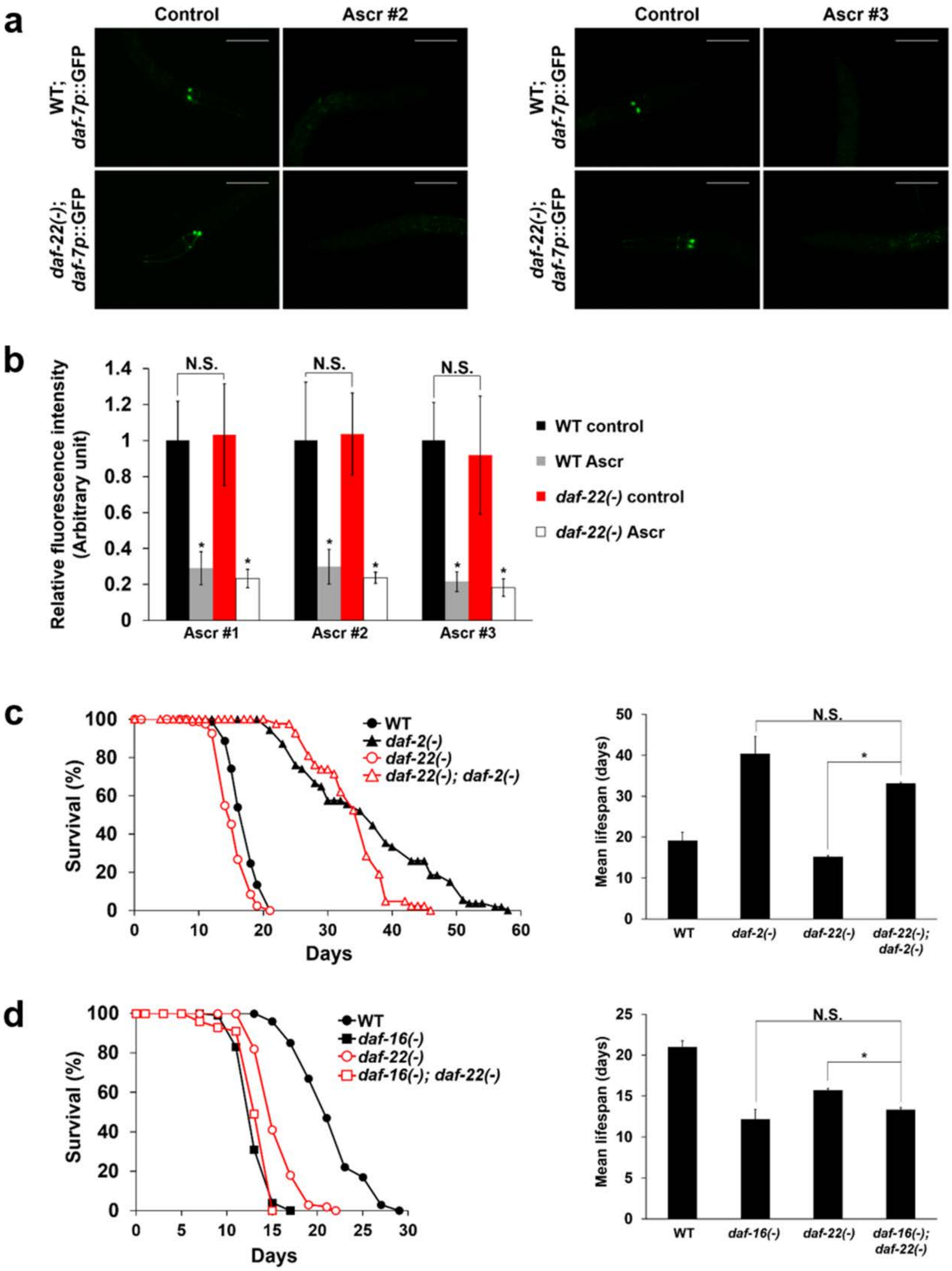

Supplementary Fig. S3. Dauer development and lifespan of *daf-22(ok693)* worms depend on IIS-governed DAF-16 activity.

(**a**) Changes of *daf-7p*::GFP expression in response to exogenous Ascr #2 (*Left*) and Ascr #3 (*Right*) in WT and *daf-22(ok693)* worms. L2-stage worms grown on either control (0 μM) or ascaroside-containing (380 μM) plates were observed. Images are views of the head region of a representative L2-stage worm. Scale bar, 50 μm. See also Fig. 3a.

(**b**) Changes of relative fluorescence intensity of *daf-7p*::GFP expression in WT and *daf-22(ok693)* worms in response to exogenous ascarosides (Ascr #1, Ascr #2, and Ascr #3 [380 μM]). Data are shown as mean ± standard deviation. N.S., not significant compared with WT worms grown on control plates (WT control), * $P < 0.05$ compared with control for each genotype (Ascr compared with control), Student's *t*-test.

(**c**) Adult lifespan of WT, *daf-2(e1370)*, *daf-22(ok693)*, and *daf-22(ok693);daf-2(e1370)* worms. (*Left*) Representative results of two independent experiments. (*Right*) Mean lifespan. Data are shown as mean ± SEM of two independent experiments. N.S., not significant, * $P <$ 0.05 compared with *daf-22(ok693);daf-2(e1370)*, Student's *t*-test.

(**d**) Adult lifespan of WT, *daf-16(mu86)*, *daf-22(ok693)*, and *daf-16(mu86);daf-22(ok693)* worms. (*Left*) Representative results of two independent experiments. (*Right*) Mean lifespan. Data are shown as mean ± SEM of two independent experiments. N.S., not significant, * $P <$ 0.05 compared with *daf-16(mu86);daf-22(ok693)*, Student's *t*-test.

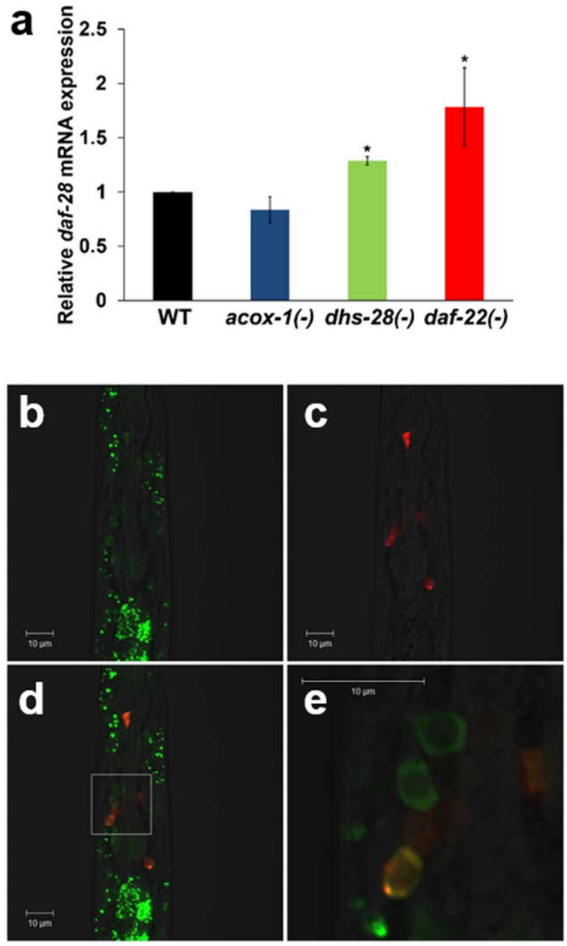

Supplementary Fig. S4. Insulin-like peptide *daf-28* expression is increased in *daf-22(ok693)* worms.

(**a**) Relative *daf-28* mRNA expression in WT, *acox-1(ok2257)*, *dhs-28(tm2581)*, and *daf-22(ok693)* worms. Data are shown as mean ± SEM of three independent experiments. * $P <$ 0.05 compared with WT worms, Student's *t*-test.

(**b-e**) Co-expression of *daf-22p*::GFP and *daf-28p*::DsRed in head neurons. Images are lateral views of the head region of a representative worm. Scale bar, 10 µm. (**b**) The expression loci of *daf-22p*::GFP::*daf-22* in intestine, hypodermis and neurons. (**c**) *daf-28p*::DsRed expression

in amphid chemosensory neurons. (**d**) Merged image of *daf-22p*::GFP::*daf-22* and *daf-28p*::DsRed expression. (**e**) Magnification of the boxed area in (d).

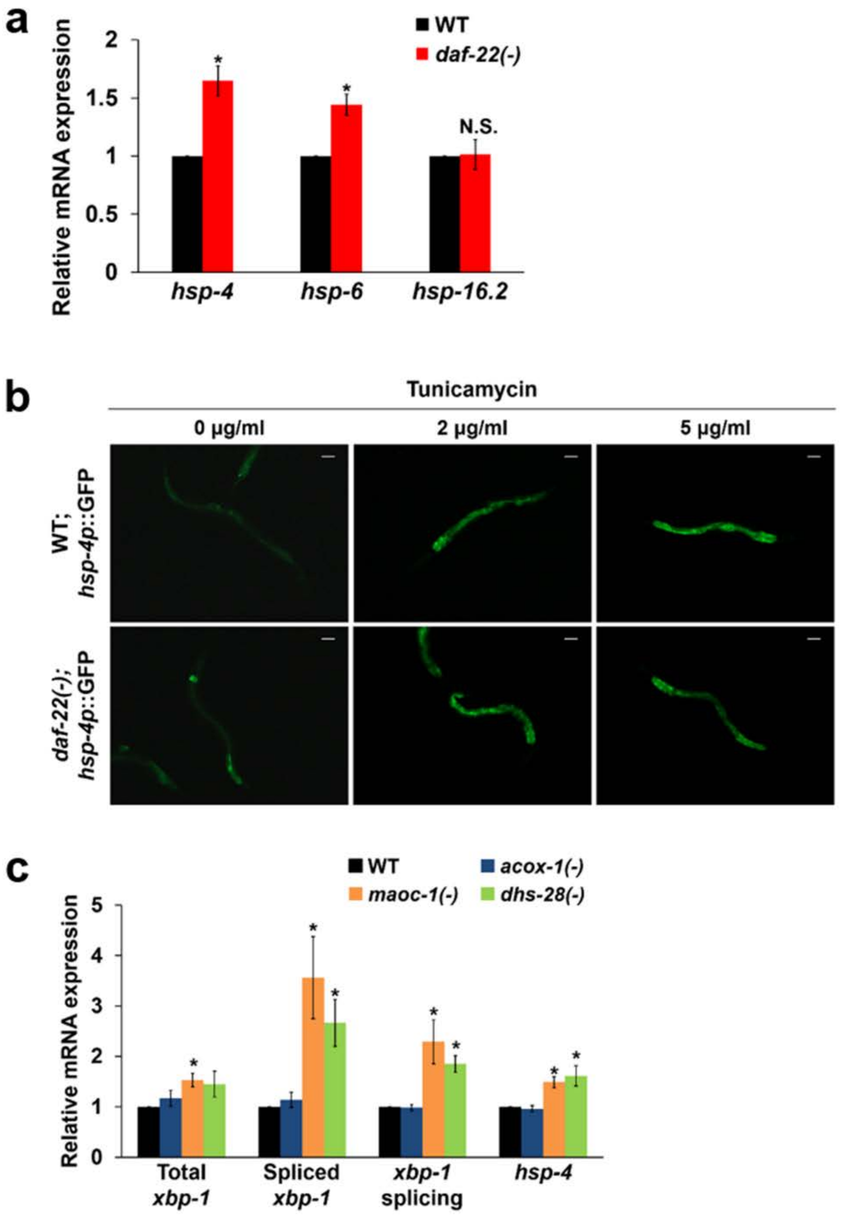

Supplementary Fig. S5. ER stress response is increased by deficiency in peroxisomal FA β-oxidation.

(**a**) Relative mRNA expression of representative stress response marker genes in young adult *daf-22(ok693)* worms compared with WT worms. *hsp-4*, ER stress marker; *hsp-6*, mitochondrial stress marker; and *hsp-16.2*, cytosolic stress marker. Data are shown as mean ±

SEM of two independent experiments. N.S., not significant, * $P < 0.05$ compared with WT worms, Student's *t*-test.

(**b**) Unfolded protein response (UPR) to proteotoxic stress in WT and *daf-22(ok693)* worms was measured by *hsp-4p*::GFP expression in response to tunicamycin treatment. Scale bar, 50 µm. Note that *hsp-4p*::GFP expression was increased in *daf-22(ok693)* worms in the absence of tunicamycin, compared to that in WT worms.

(**c**) Relative abundance of *xbp-1* splicing (spliced *xbp-1*/total *xbp-1* transcript) and *hsp-4* mRNA expression in WT, *acox-1(ok2257), maoc-1(hj13),* and *dhs-28(tm2581)* worms. Data are shown as mean ± SEM of three independent experiments. * $P < 0.05$ compared with WT worms, Student's *t*-test.

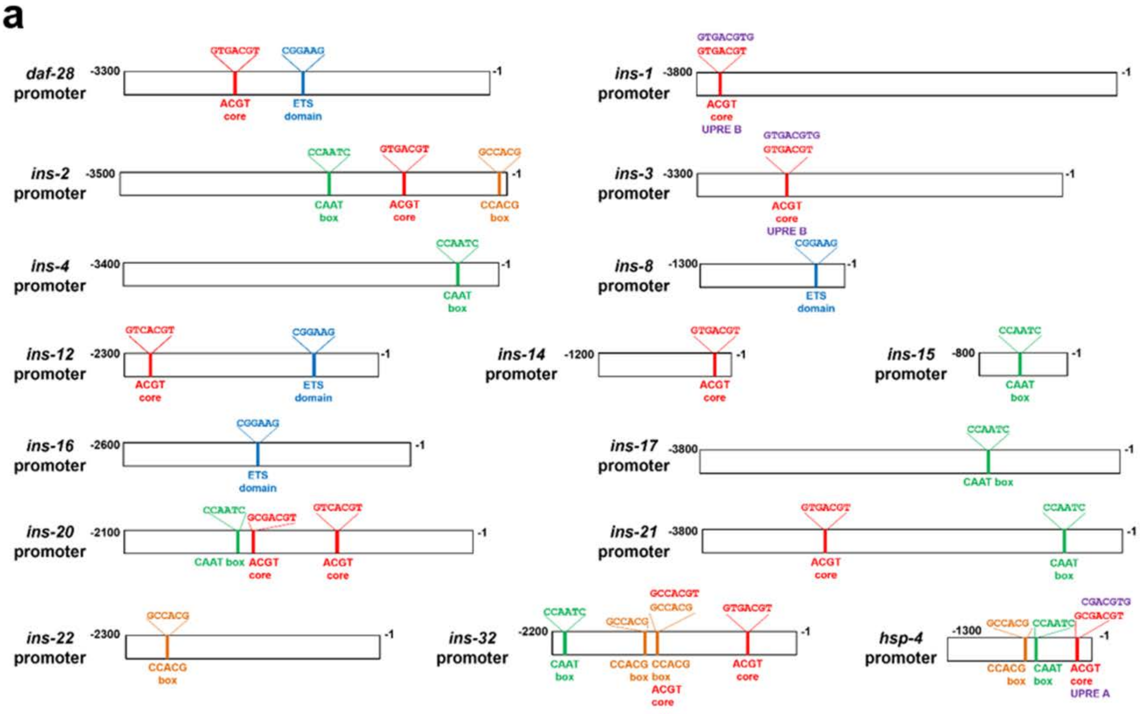

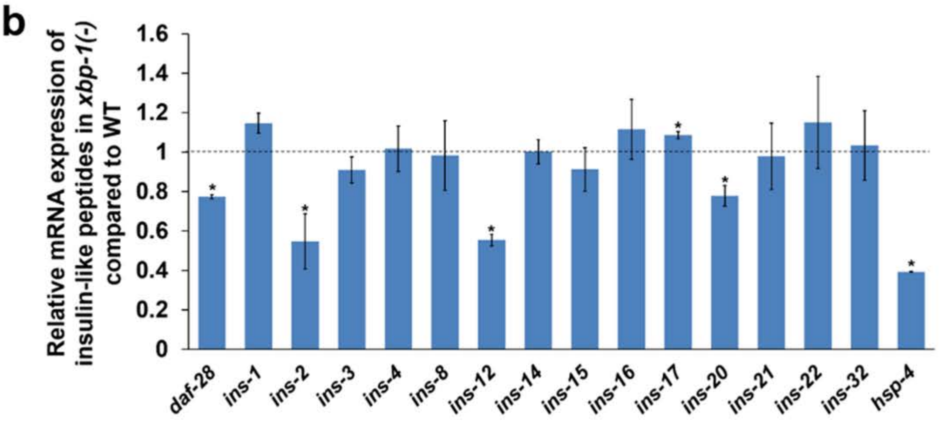

Supplementary Fig. S6. XBP-1 activity enhances expression of ILPs.

(**a**) Promoter regions of ILPs that were significantly increased in L2 *daf-22(ok693)* worms were examined for XBP-1 binding sites. *hsp-4* was examined as a control of XBP-1 binding. Several XBP-1 binding motifs in promoter regions of 15 ILPs and *hsp-4* are indicated as schematic diagrams: CAAT box (5′-CCAATC-3′), ETS domain (5′-CGGAAG-3′), CCACG

box (5′-GCCACG-3′), ACGT core (5′-G(C/T)(C/G)ACGT-3′), UPRE A element (5′-CGACGTGG-3′), and UPRE B element (5′-GTGACGTG-3′).

(**b**) Changes in relative mRNA expression of 15 ILPs with XBP-1 binding sites in their promoter regions in L2 *xbp-1(zc12)* worms. *hsp-4* mRNA expression was examined as a control. Dotted line indicates reference ILP expression in WT worms. Data are shown as mean ± SEM of two independent experiments. * $P < 0.05$ compared with WT worms, Student's *t*-test.

SUPPLEMENTARY TABLES

Supplementary Table S1. *daf-22* function in ASK neurons is not involved in ascaroside pheromone biosynthesis.

Genetic background	Promoter driving *daf-22* cDNA	Cells expressing *daf-22* cDNA	Calculated concentration (fmol/µl) (Relative fold of ascaroside concentration)		
			Ascr #1	Ascr #2	Ascr #3
**N2 (WT)**	None		59.48 ± 2.74 (1.00)	8.49 ± 0.31 (1.00)	17.59 ± 0.95 (1.00)
daf-22 (ok693)	None		0.66 ± 0.21 (0.01 ± 0.00)[a]	0.25 ± 0.13 (0.03 ± 0.02)[a]	0.41 ± 0.23 (0.02 ± 0.02)[a]
daf-22 (ok693)	*daf-22p*	Hypodermis Intestine Neuron	11.93 ± 1.34 (0.20 ± 0.01)[a,b]	13.64 ± 0.97 (1.62 ± 0.14)[b]	20.51 ± 2.07 (1.18 ± 0.13)[b]
	vha-6p	Intestine	7.12 ± 0.70 (0.12 ± 0.01)[a,b]	8.59 ± 0.39 (1.01 ± 0.05)[b]	17.81 ± 1.87 (1.01 ± 0.09)[b]
	unc-119p	Pan-neuron	5.82 ± 0.34 (0.10 ± 0.00)[a,b]	3.67 ± 0.59 (0.43 ± 0.06)[a,b]	22.05 ± 1.15 (1.25 ± 0.03)[b]
	srbc-64p	ASK	1.03 ± 0.19 (0.02 ± 0.00)[a]	0.28 ± 0.12 (0.03 ± 0.02)[a]	4.60 ± 0.89 (0.26 ± 0.05)[a]

Ascaroside pheromones (Ascr #1, Ascr #2, and Ascr #3) in worm bodies of tissue- and cell-specific *daf-22* rescue worms were measured and compared with WT and *daf-22(ok693)* worms. Data are shown as mean ± SEM of four independent experiments with three technical repeats. See also Fig. 2e, f.

[a] Statistically significant ($P < 0.001$) compared with WT worms, Student's *t*-test.

[b] Statistically significant ($P < 0.001$) compared with *daf-22(ok693)* worms, Student's *t*-test.

Supplementary Table S2. Fraction of worms that express *daf-7p*::GFP when exposed to ascarosides.

Genotype	Ascr #1		Ascr #2		Ascr #3	
	0 μM	380 μM	0 μM	380 μM	0 μM	380 μM
**WT**	42/42	0/60	43/43	0/64	77/77	2/52
daf-22(ok693)	50/50	1/36	44/44	0/30	36/36	0/53

Fraction of worms expressing *daf-7p*::GFP in response to exogenous ascaroside pheromones (Ascr #1, Ascr #2, and Ascr #3 [380 μM]). *daf-7p*::GFP expressing worms were scored. 0 μM indicates control condition. See also Fig. 3a and Supplementary Fig. S3a, b.

SUPPLEMENTARY METHODS

C. elegans strains and maintenance

The following strains were used in this study: N2 Bristol (wild-type; WT), RB859 *daf-22(ok693)* II, VC1785 *acox-1(ok2257)* I, VS18 *maoc-1(hj13)* II, FX02581 *dhs-28(tm2581)* X, VS24 *kat-1(tm1037)* II, CB1372 *daf-7(e1372)* III, CB1370 *daf-2(e1370)* III, CF1038 *daf-16(mu86)* I, FX02308 *daf-28(tm2308)* V, RB545 *pek-1(ok275)* X, RB772 *atf-6(ok551)* X, RB925 *ire-1(ok799)* II, YP801 N2;ykpIs210[*daf-22p*::GFP::*daf-22*], FK181 ksIs2[*daf-7p*::GFP + *rol-6(su1006)*], GR1455 mgIs40[*daf-28p*::GFP], SJ4005 zcIs4[*hsp-4p*::GFP] V, SJ17 *xbp-1(zc12)* III;zcIs4[*hsp-4p*::GFP] V, YP0058 *daf-22(ok693)* II;*daf-7(e1372)* III, YP0025 *daf-22(ok693)* II;*daf-2(e1370)* III, YP0018 *daf-16(mu86)* I;*daf-22(ok693)* II, YP0026 *daf-22(ok693)* II;*daf-28(tm2308)* V, YP0059 *daf-22(ok693)* II; Ex[*daf-22p*::*daf-22* cDNA + *rol-6(su1006)*], YP0060 *daf-22(ok693)* II; Ex[*vha-6p*::*daf-22* cDNA + *rol-6(su1006)*], YP0061 *daf-22(ok693)* II;ykpEx023[*unc-119p*::*daf-22* cDNA::*unc-54* 3′UTR + *rol-6(su1006)*], YP0062 *daf-22(ok693)* II;ykpEx024[*srbc-64p*::*daf-22* cDNA::*unc-54* 3′UTR + *rol-6(su1006)*], YP0063 *daf-22(ok693)* II;ksIs2[*daf-7p*::GFP + *rol-6(su1006)*], YP0064 *daf-22(ok693)* II;mgIs40[*daf-28p*::GFP], YP0023 *daf-22(ok693)* II;zcIs4[*hsp-4p*::GFP] V, YP0065 N2;zcIs4[*hsp-4p*::GFP] V;ykpEx020[*daf-28p*::DsRed + *rol-6(su1006)*], YP0066 *daf-22(ok693)* II;zcIs4[*hsp-4p*::GFP] V;ykpEx020[*daf-28p*::DsRed + *rol-6(su1006)*], YP0079 *daf-22(ok693)* II; *xbp-1(zc12)* III; zcIs4[*hsp-4p*::GFP] V, YP0084 *daf-22(ok693)* II; mgIs40[*daf-28p*::GFP]; ykpEx024[*srbc-64p*::*daf-22* cDNA::*unc-54* 3'UTR + *rol-6(su1006)*], and YP0085 N2; ykpIs210[*daf-22p*::GFP::*daf-22*]; ykpEx020 [*daf-28p*::DsRed + *rol-6(su1006)*]. Some strains were provided by the Caenorhabditis Genetics Center, which is funded by NIH Office of Research Infrastructure Programs (P40 OD010440). Mutant animals were outcrossed with N2 more than four times. RB545 and RB772 were 2× outcrossed.

Double mutant and triple mutant worms were generated using standard genetic techniques.

Cloning and transgenic-line construction

Pan-neural *daf-22* rescue transgene ykp023 [*unc-119p*::*daf-22* cDNA::*unc-54* 3′UTR] and ASK neuron-specific *daf-22* rescue transgene ykp024 [*srbc-64p*::*daf-22* cDNA::*unc-54* 3′UTR] were constructed by stitching PCR fragments of *unc-119* promoter (2.2 kb) or *srbc-64* promoter (2.0 kb), respectively, to *daf-22* cDNA (1.2 kb, reverse-transcribed from RNA) and *unc-54* 3′UTR (0.9 kb, amplified from pPD95.75). Phusion® High-Fidelity DNA Polymerase (NEB) was used for PCR. The final *unc-119* promoter::*daf-22* cDNA::*unc-54* 3′UTR and *srbc-64* promoter::*daf-22* cDNA::*unc-54* 3′UTR PCR fragments were ligated into pTOP TA V2 (TOP cloner™ TA core kit, Enzynomics). The constructed clone plasmids were linearized by restriction enzyme ScaI (NEB) to inhibit non-specific expression and were microinjected into the gonads of *daf-22(ok693)* worms at 50 ng/µl with co-injection marker pRF4 *rol-6(su1006)* (50 ng/µl) to generate the transgenic worms YP0061 *daf-22(ok693)* II;ykpEx023[*unc-119p*::*daf-22* cDNA::*unc-54* 3′UTR + *rol-6(su1006)*] and YP0062 *daf-22(ok693)* II;ykpEx024[*srbc-64p*::*daf-22* cDNA::*unc-54* 3′UTR + *rol-6(su1006)*]. *daf-28* transcriptional reporter ykp020 [*daf-28p*::DsRed] was constructed by the same method, and transgenic worms (YP0067 N2;ykpEx020[*daf-28p*::DsRed + *rol-6(su1006)*]) were crossed with SJ4005 or YP801 to generate double-transgenic mutant worms (YP0065 N2;zcIs4[*hsp-4p*::GFP] V;ykpEx020[*daf-28p*::DsRed + *rol-6(su1006)*] and YP0085 N2; ykpIs210[*daf-22p*::GFP::*daf-22*]; ykpEx020 [*daf-28p*::DsRed + *rol-6(su1006)*], respectively). The constructed transgenic worms were selected and maintained by picking rollers. Transgenic worms YP801, YP0059, and YP0060 were constructed and used in previous studies[1,2].

REFERENCES

1 Joo, H. J. *et al.* Caenorhabditis elegans utilizes dauer pheromone biosynthesis to dispose of toxic peroxisomal fatty acids for cellular homoeostasis. *Biochem. J.* **422**, 61-71, doi:10.1042/bj20090513 (2009).

2 Kim, K. Y. *et al.* Development of a method to quantitate nematode pheromone for study of small-molecule metabolism in Caenorhabditis elegans. *Anal. Chem.* **85**, 2681-2688, doi:10.1021/ac4001964 (2013).
